# Progressive pseudorheumatoid dysplasia misdiagnosed as juvenile idiopathic arthritis: a case report

**DOI:** 10.1186/s13256-021-03082-z

**Published:** 2021-11-08

**Authors:** Anjumanara Anver Omar, Salman Ahmed, John Chris Rodrigues, Allan Kayiza, Lawrence Owino

**Affiliations:** 1 Department of Paediatrics and Child Health, University of Nairobi, Nairobi, Kenya; 2 Department of Diagnostic and Imaging Radiation, University of Nairobi, Nairobi, Kenya; 3 Department of Radiology, Kenyatta National Hospital, Nairobi, Kenya

**Keywords:** Progressive pseudorheumatoid dysplasia, *WISP3* gene, Juvenile idiopathic arthritis, Bone deformity

## Abstract

**Background:**

Progressive pseudorheumatoid dysplasia is a rare, autosomal recessively inherited, noninflammatory musculoskeletal disorder caused by mutations occurring in the WNT1-inducible signaling pathway protein 3 gene. Joint cartilage is the primary site of involvement, leading to arthralgia, joint stiffness, contractures, enlargement of the epiphyses and metaphysis of the hand joints, spinal abnormalities, short stature, early osteoarthritis, and osteoporosis. Juvenile idiopathic arthritis is the most common chronic rheumatic disease in childhood and has unknown etiology. Clinical features of progressive pseudorheumatoid dysplasia resemble those of juvenile idiopathic arthritis. Patients with progressive pseudorheumatoid dysplasia are usually misdiagnosed as having juvenile idiopathic arthritis.

**Case presentation:**

A 13-year-old Yemeni female presented to the rheumatology clinic with a history of joint pains, bone pains, and bone deformity for 7 years. Weight and height were below the third percentiles. There was no tender swelling of metacarpophalangeal and interphalangeal joints, and she presented with scoliosis. Radiographs of the hands revealed the widening of the epiphyses. Progressive pseudorheumatoid dysplasia was suspected, and genetic testing for WNT1-inducible signaling pathway protein 1, 2, and 3 was requested with these findings. A homozygous, likely pathogenic variant was identified in the WNT1-inducible signaling pathway protein 3 gene, which confirmed our diagnosis.

**Conclusion:**

Progressive pseudorheumatoid dysplasia is a rare form of spondyloepimetaphyseal dysplasia and is clinically misdiagnosed as juvenile idiopathic arthritis. It is crucial to consider progressive pseudorheumatoid dysplasia, especially in patients with standard inflammatory markers who are being followed up for juvenile idiopathic arthritis and not improving with antirheumatic intervention.

## Background

Progressive pseudorheumatoid dysplasia (PPRD) is a joint disease that worsens over time. This condition is characterized by the degeneration of the articular cartilage covering and protecting the ends of bones [1].

Progressive pseudorheumatoid dysplasia has been estimated to occur in approximately 1 per million people in the UK. Though this condition is thought to be more common in Turkey and the Middle East, its prevalence is unknown [2]. The situation in all regions is likely underdiagnosed because it is often misdiagnosed as juvenile idiopathic arthritis (JIA) [3]. PPRD usually begins in childhood, between ages 3 and 6 years [1, 4]. The first indications are generally interphalangeal joint swelling, pain, abnormal walking pattern plus weakness and fatigue when active, and stiffness in the fingers and knee joints [5]. Other signs and symptoms that develop over time include permanently bent fingers (camptodactyly), enlarged finger and knee joints (often mistaken as intraarticular swelling), and a reduced amount of space between the bones at the hip and knee joints. Hip pain is a common problem in adolescence. Affected individuals have flattened bones in the spine (platyspondyly) that are abnormally shaped (beaked), which leads to an abnormal front-to-back curvature of the spine (kyphosis) and a short torso [1]. People with PPRD are of standard length at birth, but they are usually shorter than their peers [5].

This disorder is caused by a loss of function of the *WISP3* gene (WNT1-inducible signaling pathway protein 3; MIM#603400) encoded on chromosome 6q21. WISP3 is essential in maintaining cartilage integrity by regulating the expression of type II collagen and aggrecan in chondrocytes. Therefore, it is crucial for bone formation and maintaining cartilage [4–6]. The clinical features are similar to those of JIA, and patients with PPRD are usually misdiagnosed as having JIA [3]. There is not enough literature on cases with PPRD in Sub-Saharan Africa, mainly due to misdiagnosis or lack of knowledge on rare diseases. Even though it is more prevalent in Arab countries owing to the high prevalence of consanguinity, it can still occur in African countries where consanguineous marriages are minimal. Therefore, it was prudent to highlight this case so that such cases like hers are not missed in the future.

We present this case report of a 13-year-old girl who followed up for JIA for 4 years.

## Case presentation

We report a case of a 13-year-old Yemeni female who was referred to the rheumatology clinic with a history of joint pains, bone pains, and bone deformity for 7 years. She was born via spontaneous vaginal delivery without complication to consanguineous parents of Yemeni origin. She is the third born in her family. There is no history of joint disorders in the family. Joint pains and bone pains started at 6 years of age. This was followed by easy fatigability and swelling of the proximal (PIP) and distal interphalangeal joints (DIP), followed by the elbow joints. This was accompanied by pain in the left hip, causing her to limp, and finally the inability to walk.

**Fig. 1:**
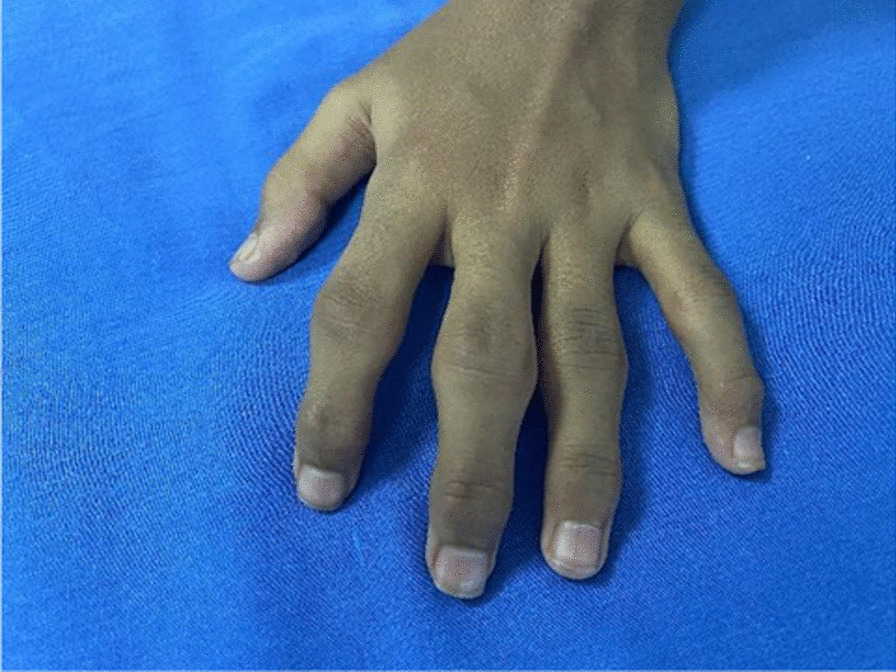
Swelling of the proximal distal interphalangeal joints of the left hand

**Fig. 2:**
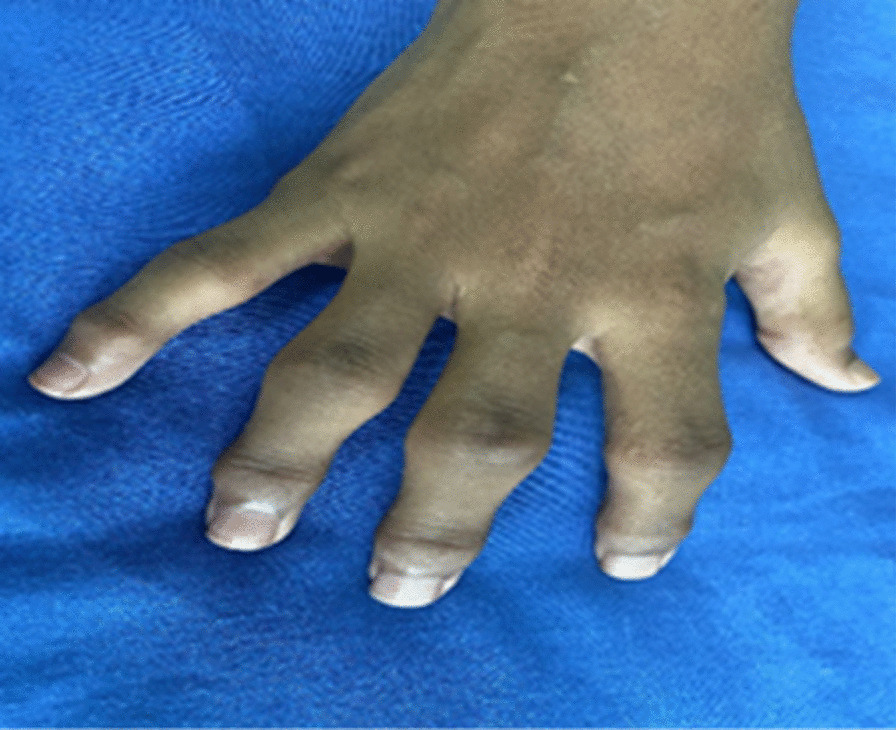
Swelling of the proximal and distal interphalangeal joints of the right hand; non-erythematous and non-tender

Initially, at the age of 9 years, she presented to the general practitioner with joint and bone pains. She was diagnosed with juvenile idiopathic arthritis and treated with non steroidal anti-inflammaroty drugs (NSAIDS). However, her condition kept deteriorating despite the NSAIDS for 4 years, and she was referred to a pediatric rheumatologist for further management. Her weight and height on presentation were below the third percentile, and upper-to-lower body segment ratio was 1. On examination, the patient was in generally fair condition. She had marked prominence (swelling) of proximal (PIP) and distal interphalangeal joints (DIP) bilaterally, which were non-erythematous and non-tender (Figs. 1,2). However, there was limited mobility of the joints. Swelling of the knee and elbow joints bilaterally was also noted. She also had scoliosis on presentation.

She was prepubertal at 13 years of age. Neurological examination did not reveal any abnormal findings. Radiographs of the hands and spine were requested.

## Radiological assessment

**Fig. 3:**
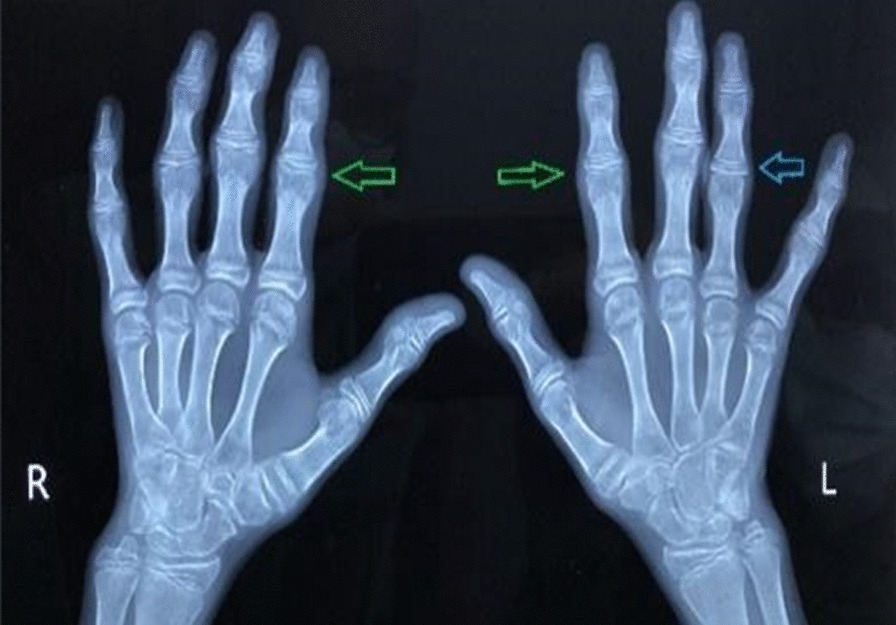
Reduced joint space between proximal interphalangeal joints. Arrows indicate increased soft tissue density between the proximal interpharyngeal joints

**Fig. 4:**
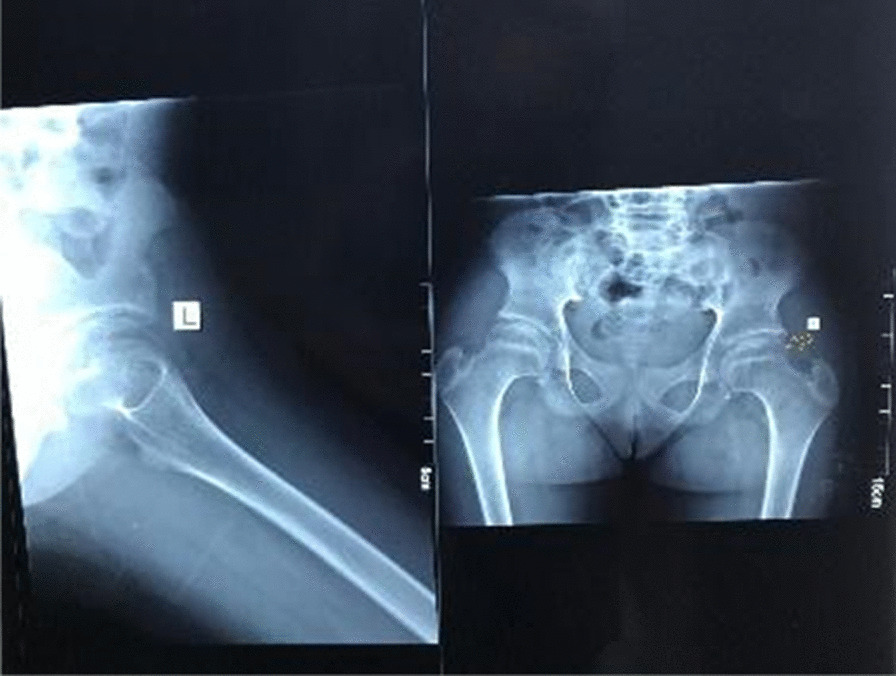
Reduction in the left femoral epiphysis, with sclerosis

**Fig. 5:**
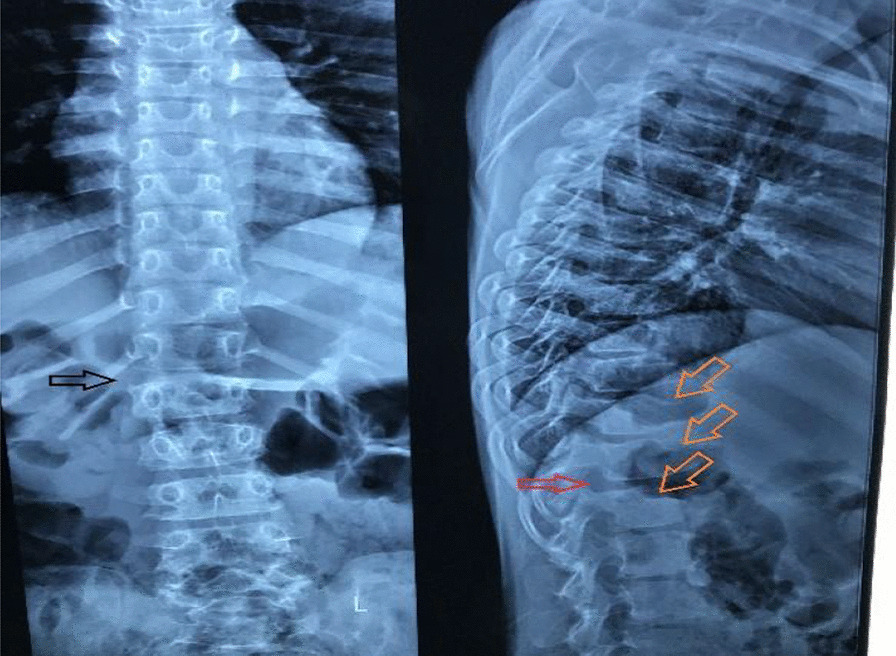
Anterosuperior endplate compression of the vertebra. Black arrow shows reduced inter-vertebral disk space and platyspondyly, red arrow shows dorsal osteopenia, orange arrows show anterior degradation of the vertebral bodies

Radiology of the hands and spine was taken and revealed the following findings: reduced joint space in the third and fourth proximal interphalangeal joints with increased soft tissue density around the proximal interphalangeal joints (Fig. 3) Radiology of the hip revealed reduction in the left femoral epiphysis, with associated mild sclerosis. A left femoral epiphyseal subchondral lucency presence was consistent with avascular necrosis of the left femoral head (Fig. 4). A radiograph of the thoracolumbar spine revealed mild scoliosis with loss of height of the lower thoracic and upper lumbar vertebral bodies. Anterosuperior endplate compression changes of multiple vertebrae were also noted (Fig. 5).

## Diagnosis

Given the clinical symptoms, laboratory tests that included biological markers of inflammation and complete blood count, were requested. These are summarized in Table 1 below.

**Table 1:** Biological markers and complete blood count

Parameter	Result	Reference range
HLA B27	Not detected	
ANA	Negative	
RF	< 9.5	Negative: < 30 Positive: > 30
ESR	5 mm	0–20 mm
CRP	Negative	
Complete blood count		
Hemoglobin	13.0 g/dl	11.5–15.5 g/dl
White blood cell count	8.15 × 10^3^/mm^3^	5–14.5 × 10^3^/mm^3^
Neutrophils	54%	33–76%
Lymphocytes	38%	15–61%
Platelets	340 × 10^3^/mm^3^	150–450 × 10^3^/mm^3^

HLA B27, Human leukocyte antigen B27; ANA, Anti-Nuclear antibody; RF, Rheumatoid Factor; ESR, Erythrocyte sedimentation rate; CRP, C-reactive protein

Because of the deterioration of the patient’s condition, and the laboratory and radiologic findings that were not in keeping with the diagnosis of JIA, PPRD was strongly suspected. Therefore, a genetic analysis of the *WISP3* gene was carried out. Double-stranded Deoxyribonucleic acid (DNA) capture baits against approximately 36.5 Mb of the human coding exome were used to enrich target regions from fragmented genomic DNA with the Twist Human Core Exome Plus kit. The generated library was sequenced to obtain at least ×20 coverage depth for > 98% of the target bases; then a bioinformatics pipeline was applied. All disease-causing variants below 1% in the gnomAD database were considered. The investigation for relevant variants was focused on coding exons and flaking ±20 intronic bases. All potential modes of inheritance patterns were considered. Besides, provided family history and clinical information were used to evaluate identified variants concerning this pathogenicity and causality, categorized as pathogenic, likely pathogenic, variant of uncertain significance, likely benign, or benign. A homozygous possible pathogenic variant was identified in the *WISP3* gene, a novel compound mutation in the WISP3 variant c.746delT p. (Val249Glyfs*10) creating a premature stop codon nine positions downstream. There was no detection of pathogenic or likely pathogenic variants in the genes for which incidental findings were reported. The parents declined to have the test, so they were not assessed.

Once the diagnosis of PPRD was made, she was started on NSAIDs for pain relief, and a recommendation was also made for joint replacement surgery. She is on follow-up, and there has been a marked reduction in the pain, though the deformity persists.

## Discussion and conclusion

PPRD is characterized by the predominant involvement of articular cartilage with progressive joint stiffness and enlargement in the absence of inflammation. Starting between ages 3 and 6 years with the involvement of the interphalangeal joints as the initial clinical presentation [5] and, over time, involving the large joints and the spine, it causes significant joint contractures, gait abnormality, scoliosis, or kyphosis, which results in abnormal posture and considerable morbidity [5]. Likewise, our patient initially presented to the general practitioner with complaints of swelling of the proximal interphalangeal joints bilaterally. Like in our patient, in all regions, this condition is likely to be underdiagnosed because it is often misdiagnosed as juvenile idiopathic arthritis (JIA). Our patient had been on follow-up for JIA for 4 years and had been put on NSAIDS with no improvement. The disease progressed to the hip joints, causing immobility. PPRD is differentiated from JIA by the absence of inflammation, extraskeletal manifestations, and articular bone erosion.

Moreover, these patients do not respond to antirheumatic agents and therefore will not yield any clinical benefit. Our patient was put on painkillers and was advised on hip replacement surgery. There is no cure for PPRD, but treatment may include pain medication and hip and knee joint replacement surgery at an early age [5]. The radiological features of JIA are different from PPRD, including spondyloepiphyseal dysplasia with platyspondyly as an early finding and lack of destructive joint erosions [7, 8].

Although radiologic examination has high accuracy in diagnosing PPRD, the definitive diagnosis is established in a proband with identifying the characteristic radiologic findings and biallelic pathogenic variants in the *WISP3* gene on molecular genetic testing [5]. In our patient, a novel compound homozygous mutation in the *WISP3* gene was identified. Genetic testing and counseling should be offered to these families to plan for the next generation.

Medical management of PPRD remains symptomatic, and hip joint replacement surgery in adolescents is recommended. In this case, our patient’s pain was controlled with NSAIDs, and her parents plan for surgery in the future.

In conclusion, patients presenting with musculoskeletal symptoms should be evaluated appropriately to prevent inappropriate therapy, and to start appropriate therapy in time.

## Data Availability

Data sharing does not apply to this article as no datasets were generated or analyzed during the current study

## Abbreviations

ANA: Antinuclear antibody
DIP: Distal interphalangeal
HLA-B27: Human leukocyte antigen
JIA: Juvenile idiopathic arthritis
NSAIDs: Nonsteroid antiinflammatory drugs
PIP: Proximal interphalangeal
PPRD: Progressive pseudorheumatoid dysplasia
RF: Rheumatic factor
WISP3: WNT1-inducible signaling pathway protein 3
